# Effects of Clenching Strength on Step Reaction Time

**DOI:** 10.3390/jfmk10030264

**Published:** 2025-07-13

**Authors:** Nao Sugai, Ryo Hirabayashi, Yoshiyuki Okada, Yuriko Yoshida, Takeru Okouchi, Hirotake Yokota, Tomonobu Ishigaki, Makoto Komiya, Mutsuaki Edama

**Affiliations:** 1 Institute for Human Movement and Medical Sciences, Niigata University of Health and Welfare, 1398 Shimami-cho, Kita-ku, Niigata-shi, Niigata 950-3198, Japan; hpm24002@nuhw.ac.jp (N.S.); hpm23013@nuhw.ac.jp (T.O.); yokota@nuhw.ac.jp (H.Y.); tomonobu-ishigaki@nuhw.ac.jp (T.I.); makoto-komiya@nuhw.ac.jp (M.K.); edama@nuhw.ac.jp (M.E.); 2 Graduate School of Biomedical and Health Sciences, Hiroshima University, Hiroshima 734-8551, Japan; okay@hiroshima-u.ac.jp (Y.O.); yoshiyu@hiroshima-u.ac.jp (Y.Y.)

**Keywords:** clenching, remote muscle activation, electromyogram, step action, remote facilitation effect

## Abstract

**Background:** Reaction time is analyzed in various situations in sporting events and is reported to be so important that it can make the difference between victory and defeat. This study focused on teeth clenching resulting in remote muscle activation, and examined whether it improves performance of reaction time. This study examined the effects of clenching and clenching strength on the systemic simple reaction time. **Methods:** This study included 20 healthy adults with normal clenching and a right dominant foot. The task movement for the systemic simple reaction time measurement was a 30 cm forward step. The following three clenching conditions were used: no clenching without dental contact (no-bite condition), a condition in which the participants were instructed to clench with moderate strength (moderate condition), and a condition in which the participants clenching with maximum effort (max condition). The analysis items were release time, grounding time, soleus muscle (Sol) reaction time, and masseter muscle activity. **Results:** The max condition significantly reduced the reaction time compared with the no-bite condition. Sol reaction and grounding times showed a negative correlation between clenching strength under moderate conditions and the rate of change in reaction time under no-bite and moderate conditions. Release time exhibited no significant correlation between clenching strength under the moderate condition and the rate of change in reaction time under the no-bite and moderate conditions. The remote facilitation effect of clenching improved the systemic reaction time by producing immediate muscle activity. **Conclusions:** Clenching shortens the systemic simple reaction time. This finding highlights the potential importance of clenching in enhancing performance during sporting events.

## 1. Introduction

Reaction time is analyzed in many situations in sports, and is an important factor for improving sports performance [1,2]. Reaction time can be assessed for various types, such as simple reaction time, selective reaction time, reaction inhibition, anticipatory reaction, and reflexive reaction [2]. Among these reaction capabilities, we focused on the simple reaction time. Simple reaction time is defined as the time it takes an individual to respond to an external stimulus. Because the action to the external stimulus is predetermined, the action can be performed immediately after the stimulus [3,4]. Examples include athletics, swimming, and motorsport, where the start is made simultaneously as the signal, and where shortening the simple reaction time can make or break a race [5,6,7]. Motor commands from the brain are necessary to respond to external stimuli. This motor command involves motor-related areas in the brain [8,9]. For example, when visual information is received, the motor-related areas are as follows: visual information is perceived in the visual cortex of the occipital lobe and is processed in the temporal and parietal cortices to generate a body diagram. The generated body schematic is sent to the supplementary motor cortex and premotor cortex, which generate motor programs and transmit them to the primary motor cortex. The primary motor cortex then issues motor commands through the corticospinal tract based on the motor program, and the desired movement can be performed [10]. Therefore, simple responses can generate motor programs before stimulus initiation and do not require movement selection, response control, or other demanding cognitive functions [11,12]. Thus, simple responses can produce motor commands from the primary motor cortex immediately after stimulation. Therefore, the activation of the pathways from the primary motor cortex to muscle contraction (increased excitability of the primary motor cortex, activity of corticospinal tracts, and increased spinal excitability) shortens simple reaction times [13,14,15].

We focused on teeth clenching to shorten the systemic simple reaction time. Clenching has an immediate and significant impact on athletic performance, including increased remote muscle activity, increased joint exertion torque, and improved jumping ability, which has been confirmed during all sporting activities [16,17,18,19,20]. Remote muscle activation by clenching involves a remote prompting effect [21]. The mechanism underlying the remote facilitation effect is pressure stimulation to the periodontal ligament receptors in the teeth by clenching and the firing of muscle spindles of masticatory muscles cause afferent trigeminal nerve input to the reticular formation located in the brainstem [22]. Inhibition of Ia inhibitory interneurons from reticular formation via the reticulospinal tract causes presynaptic inhibition and increases the excitability of anterior horn cells in the spinal cord [21,23]. Furthermore, the excitability of anterior horn cells of the spinal cord increases with clenching intensity [16]. Because increased excitability of anterior horn cells of the spinal cord increases mobilization of muscle motor units, clenching may cause the simultaneously firing of many motor units, resulting in high muscle exertion [24,25,26]. These results suggest that clenching shortens the muscle reaction time and the systemic simple reaction time. However, although increased clenching strength increases the excitability of anterior horn cells of the spinal cord, left–right clenching imbalance exerts a disproportionate effect on the left–right afferent trigeminal input and on the effect of remote prompting [24].

Therefore, this study aimed to investigate the effects of clenching and clenching strength on the systemic simple reaction time. The hypothesis is that clenching increases the excitability of anterior horn cells of the spinal cord as a remote facilitation effect, resulting in increased muscle activity in the lower extremities and shortened systemic simple reaction time due to the mobilization of many motor units. Furthermore, the reaction time may be shorter in participants with higher clenching strength.

## 2. Materials and Methods

### 2.1. Subject

This study included 20 right-handed healthy adults (10 males and 10 females, age: 20.8 ± 0.7 years, height: 163.8 ± 9.6 cm, weight: 55.6 ± 8.7 kg) with a normal occlusal relationship and a current number of at least 28 teeth. The study participants were determined to have normal occlusal relationship based on a occlusal evaluation by a dentist. The criteria for a normal occlusal relationship were as follows: angle class I, overbite (OVB) < 5 mm, overjet (OVJ) < 5 mm, median deviation (MLD), dental crowding, and no missing teeth. This study was approved by the Ethics Committee of Niigata University of Health and Welfare (18363-200210, 10 February 2020). This study was conducted according to the ethical standards of Niigata University of Health and Welfare and the 1964 Declaration of Helsinki and subsequent amendments.

**Figure 1:**
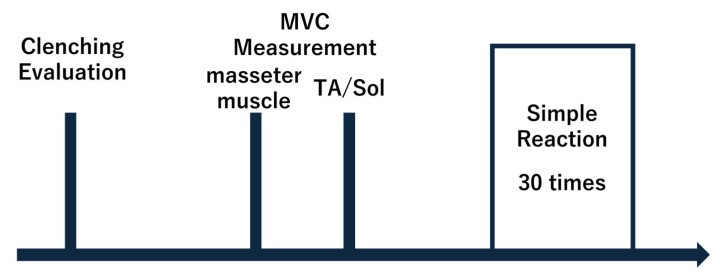
Experimental protocol. The occlusal function evaluated whether the occlusal relationship was met, and MVC measurements of the bilateral masseter and soleus muscles were performed after EMG. In the reaction time measurement, the three clenching conditions were performed five times for each of the left and right legs (30 times in total). In the systemic simple reaction time measurement, the clenching condition and step leg were indicated in advance before the measurement.

Experimental procedure (Figure 1).

A dentist evaluated the subjects to determine whether they had normal occlusion. Electromyography (EMG) was then performed on the masseter muscle (MM) bilaterally, and maximum voluntary contraction (MVC) measurements were performed. After the MVC measurements, the participants practiced the motor task six times (3 conditions × 2 times), including the three clenching conditions, using a light stimulus as a cue. Reaction time measurements were then performed 30 times (3 conditions × 5 times × 2 legs). Before measuring the reaction time, the participants were instructed on the clenching condition and step leg. All clenching conditions were randomized to avoid fatigue and order effects.

### 2.2. Electromyography Measurement (EMG)

EMG measurements were performed using a wireless system (DELSYS Trigno, 4Assist, Tokyo, Japan). The conductive surface area of the electrode was 37 (W) × 26 (D), and the fixed electrode distance was 10 mm. The electrodes were attached to the muscle bellies of the masseter muscle (MM) and soleus muscle (Sol). To identify the Sol, the subjects lay prone on a bed and plantar flexion of the ankle joint was performed with the knee joint flexed, and subjects were instructed not to overcome the examiner’s resistance. To identify the MM, the mandible and zygomatic bone were palpated, and the subjects were instructed to bite down to confirm whether the muscle belly was located at the midpoint between the mandible and zygomatic bone. Before attaching each electrode, the skin at the electrode attachment site was wiped with alcohol swabs to reduce electrical resistance. EMG electrodes were attached to the Sol and MM so that the short axis of the EMG crossed the muscle fibers perpendicularly, according to SENIAM [27]. The electromyogram waveform was processed with a 20–450 Hz bandpass filter to analyze the onset of muscle contraction in the Sol, and the sampling frequency was 2000 Hz, which was digitally stored on a personal computer. For bilateral MM MVC measurements, 8 mm cotton rolls were placed on the bilateral canines to the third molars to uniformly measure the clenching contact area on the left and right sides [28,29]. MVC measurement was instructed to be performed for approximately 3 s at maximum effort. Data analysis was performed using PowerLab 8/30 and LabChart 8 (both AD Instruments, Colorado Springs, CO, USA) after full-wave rectification of the EMG raw waveforms of the MM.

### 2.3. Clenching Conditions

Three clenching conditions were used: no clenching without dental contact (no-bite condition), clenching with moderate strength (moderate condition), and clenching with maximal effort (max condition).

**Figure 2:**
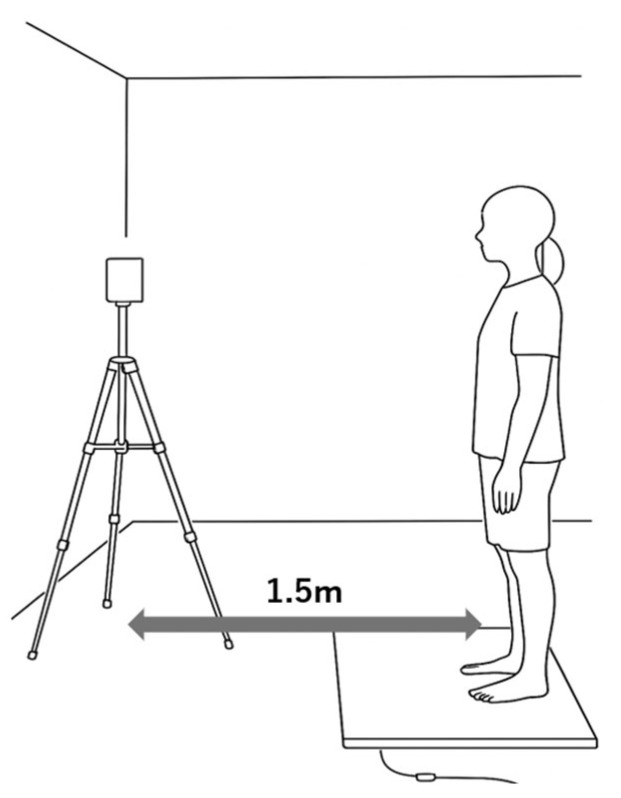
Reaction time measurement. The starting position was a standing posture at rest. The light stimulus was placed at the height of the participant’s eyes and positioned 1.5 m in front of them.

Reaction time measurement (Figure 2).

The reaction time was measured using the multitime measurement system FTM series (4Assist, Tokyo, Japan). A 5-channel time counter was used, and the bandpass was recorded on a personal computer at 10–850 Hz. Four mat switches were used, and an LED indicator was placed 150 cm in front of the participant for the presentation of light stimuli. The height of the light stimulus was set at the height of the participant’s eyes.

The exercise task was to step forward for 30 cm. The measurement position was a comfortable static standing posture with the feet 10 cm apart and the arms relaxed at the sides of the body [30,31]. The participants were instructed in advance on the clenching conditions and step legs. After clenching at the examiner’s instruction, the participant performed a forward-stepping movement guided by a light stimulus presented randomly within 2.5–3.5 s. To accurately measure reaction times, the participants were instructed not to make predictions. To avoid muscle fatigue and maintain concentration, a 1 min break was set every six exercise tasks, and the clenching condition was performed randomly.

### 2.4. Data Analysis

The analysis items were the Sol reaction time, release time, grounding time, and muscle activity of the MM. The Sol reaction time was analyzed from the EMG signal recorded from that muscle. Resting muscle activity was calculated using the standard deviation of Sol muscle activity during 0.1 s before light stimulation × 3 + mean, and the time point was defined as the time point when the muscle activity during the step exceeded the resting muscle activity. The step leg release time was defined as the time from the light stimulus cue until the foot left the mat. The grounding time was defined as the time from the light stimulus cue until the foot left the mat and was grounded to the mat in front of the foot. For the MM, %MVC was calculated by dividing the mean EMG value by MVC for the 0.1 s period before light stimulation to avoid noise.

Furthermore, the rate of change was calculated as follows: [(each clenching condition − no-bite condition)/no-bite condition × 100]. Negative values for the rate of change in the reaction time indicate shorter reaction times under the moderate conditions compared with those under the no-bite condition.

### 2.5. Statistical Analysis

The sample size was calculated using G*Power software 3.1 (Heinrich Heine University, Dusseldorf, Germany). With a significance level of 5% and a power of 80%, the sample size requirement was met with 20 subjects.

All data were statistically analyzed using the Statistical Package for the Social Sciences (v. 24; IBM, Armonk, NY, USA). The Shapiro–Wilk test was performed to test normality. Repeated measures one-way analysis of variance was performed to compare the reaction times between and within the clenching conditions. The post hoc test tests were Bonferroni corrections to the paired *t*-tests as multiple comparison tests.

Data for the comparison of reaction times between clenching conditions were averaged for the left and right legs of each participant. Spearman’s rank correlation coefficient was used for data that did not follow normality to examine the relationship between the amount of MM activity under the moderate condition and the change in reaction time under the no-bite and moderate conditions. The significance level was set at 5% in both cases.

## 3. Results

The masseter %MVC under each clenching condition was 1.3% ± 0.1% and 16.5% ± 0.2% under the no-bite and moderate conditions, respectively.

### Comparison Between the Clenching Conditions

**Figure 3:**
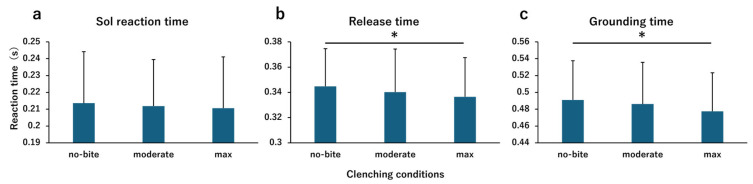
Comparison of reaction times between clenching conditions. This figure compares the reaction times between the clenching conditions. (**a**) Soleus muscle reaction time. (**b**) Release time. (**c**) Grounding time. The vertical axis of the bar graph shows the reaction time (s), indicating that the reaction time decreases as one moves downward. The horizontal axis shows, from left to right, the clenching conditions of the no-bite, moderate, and max conditions. To compare the no-bite condition with the moderate and max conditions, repeated measures one-way analysis of variance was performed, followed by Bonferroni correction. The horizontal line indicates the values that were significantly different compared with the no-bite condition (* *p* < 0.05).

Figure 3 presents the results of the comparison between the clenching conditions.

Repeated measures one-way analysis of variance revealed that the clenching condition had no main effect on the Sol reaction time (F [2,38] = 0.304, *p* = 0.739, η2 = 0.016, detection power (1 − β) = 0.095) (Figure 3a). The clenching condition exhibited a main effect on the release time (F [2,38] = 4.050, *p* = 0.025, η2 = 0.176, detection power (1 − β) = 0.686), and the clenching condition exhibited a main effect on the grounding time (F [2,38] = 6.062, *p* = 0.005, η2 = 0.242, detection power (1 − β) = 0.859). The post hoc test results revealed that the release time was significantly shorter under the max condition than under the no-bite condition (*p* = 0.019) (Figure 3b). The grounding time was significantly shorter under the max condition than under the no-bite condition (*p* = 0.004) (Figure 3c).

**Figure 4:**
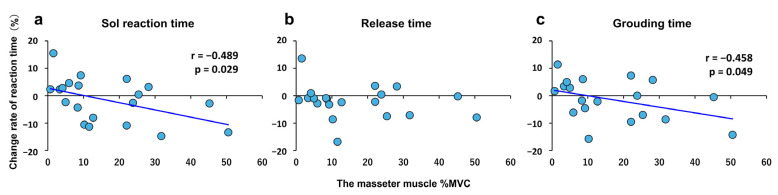
Relationship between masseter muscle activity and the rate of change in reaction time. Figure 4 shows the relationship between the amount of masseter muscle activity under the moderate condition and the rate of change in reaction time under the no-bite and moderate conditions. (**a**) Soleus muscle reaction time. (**b**) Release time. (**c**) Grounding time. The vertical axis of each graph presents the percent change in reaction time between the no-bite and moderate conditions (%). The horizontal axis shows the masseter muscle %MVC under the moderate condition for each participant (%).

Relationship between clenching strength and rate of change in reaction time under the moderate condition (Figure 4).

The Sol reaction and grounding times exhibited a negative correlation with clenching strength under the moderate condition and the rate of change in reaction time under the no-bite and moderate conditions (*r* = −0.489, *p* = 0.029 and *r* = −0.458, *p* = 0.049, respectively). No significant correlation was observed between the release time and clenching strength under the moderate condition and between the release time and the rate of change in reaction time under the no-bite and moderate conditions.

## 4. Discussion

The main findings of this study were that the max condition reduced the reaction time compared with the no-bite condition. Furthermore, the reaction time was shorter under the moderate condition than under the no-bite condition for those with higher clenching strength. Therefore, the remote prompting effect of clenching reduces the reaction time at high clenching strengths.

### 4.1. Comparison Between the Clenching Conditions

The time to leave the ground and the grounding time were significantly shorter under the max condition than under the no-bite condition. The remote prompting effect of clenching is believed to be a major factor [16,21]. The remote prompting effect has been reported to be involved in improving immediate motor performance [16]. Furthermore, increased excitability of anterior horn cells in the spinal cord increases the rate of muscle exertion and shortens the time from the onset of muscle activity to the onset of joint movement [24,26,32,33,34,35,36]. Suggesting that clenching increases the excitability of anterior horn cells in the spinal cord at a broad spinal level through a remote facilitation effect, which shortens the time to leave the ground with generalized joint movement. In contrast, despite the reduction in the systemic reaction time, no significant reduction in the Sol reaction time was observed. Because previous studies have shown that the Sol H-reflex amplitude values, a measure of spinal excitability, also increase with increasing clenching strength [16], we hypothesized that the Sol reaction time would also decrease in this study. However, contrary to this hypothesis, no significant difference was found. This may be because the Sol is a postural support muscle [37,38]. The starting limb position for this experiment was the resting standing position. Therefore, there may have been inter-participants variability in Sol muscle activity at rest, with a concomitant increase in the variability at the onset of muscle activity, which may have resulted in the lack of statistical significance.

### 4.2. Relationship Between Clenching Strength and Rate of Change in Reaction Time Under the Moderate Condition

The Sol reaction and grounding times were shorter in participants with higher clenching strength. However, a comparison of the reaction times between the clenching conditions exhibited no significant reduction in the reaction time under the moderate condition. This may be attributed to the relationship between clenching strength and increased excitability of anterior horn cells in the spinal cord. The excitability of anterior horn cells in the spinal cord increases with increasing clenching strength [16,24]. Under the moderate condition, the amount of MM activity varies among participants. Therefore, the amount of pressure stimulation to the periodontal ligament receptors of the teeth and the firing frequency of the muscle spindles of the mouth-closing muscles may have been changed by the difference in clenching strength, resulting in a change in the trigeminal nerve input. Furthermore, because the relationship between increased spinal excitability and increased muscle exertion rate has been reported in individuals with increased spinal excitability [14], in this study, people with higher clenching strength may have been more likely to activate the Sol, the primary action muscle of the step, resulting in shorter Sol reaction times, which in turn resulted in shorter grounding times. In conclusion, clenching strength is strongly related to the excitability of anterior horn cells in the spinal cord, and high clenching strength decreases the systemic reaction time. Therefore, in sporting events where shortening the reaction time is important, pre-clenching can increase performance; therefore, the findings of this study should be validated in motor tasks reflected in sporting events.

### 4.3. Study Limitations

One limitation of this study was the effect of the starting limb position during the motor task on the reaction time. The starting limb position for this study was the static standing posture, as in previous studies. The reason why this study used EMG and muscle reaction time measurements in the Sol was because our research group found a relationship between clenching strength and the excitability of anterior horn cells in the spinal cord [16]. The muscular exertion of the Sol is most likely to occur in the flexed position of the knee joint [39]. The static standing posture is a relaxed position with the knee joint in extension, which may make it difficult for the Sol to exert the muscles. Although the main active muscle in the forward stepping task is considered to be the Sol, the fact that it is a systemic task suggests that not only the ankle joints but also the knee joint and hip flexor muscles are highly involved in the task. The starting limb position, EMG measurements at the Sol, and muscle reaction time measurements in this study were performed as previously described. Therefore, EMG of the knee joint and hip flexor muscles was not performed; thus, the affects could not be examined. In the future, the effects of posture and muscle activity on other joints should also be examined.

## 5. Conclusions

This is the first study to report that clenching exerts significant effects on the systemic simple reaction time. Clenching activated remote muscles and increased the speed of systemic reactions, such as the release and grounding times. Furthermore, high clenching strength was associated with the activity of the main action muscles in the whole-body response involving joint movements. Therefore, spreading the importance of clenching and clenching strength to improve sports performance is necessary. For athletic events, such as track and field, where a reduction in the systemic simple reaction time is directly associated with athletic performance, clenching can help improve athletic performance and may be useful in various sports events. Because this study only examined the effects of basic movements, the effects of clenching on competition movements should be examined in the future.

## Data Availability

The raw data supporting the conclusions of this article will be made available by the authors upon reasonable request.
